# Spontaneous Hinge-Bending Motions of Angiotensin I Converting Enzyme: Role in Activation and Inhibition

**DOI:** 10.3390/molecules25061288

**Published:** 2020-03-12

**Authors:** Thi Tuong Vy, Seong-Yeong Heo, Won-Kyo Jung, Myunggi Yi

**Affiliations:** 1 Interdisciplinary Program of Biomedical, Electrical & Mechanical Engineering, Pukyong National University, Busan 48513, Korea; phanvy120690@gmail.com (T.T.V.); hsyadsl@naver.com (S.-Y.H.); wkjung@pknu.ac.kr (W.-K.J.); 2 Department of Biomedical Engineering, Pukyong National University, Busan 48513, Korea; 3 Marine Integrated Bionics Research Center, Pukyong National University, Busan 48513, Korea

**Keywords:** angiotensin converting enzyme, spontaneous conformational change, activation and inhibition mechanism, MD simulation, hinge-bending motion

## Abstract

The inhibition of human angiotensin I converting enzyme (ACE) has been regarded as a promising approach for the treatment of hypertension. Despite research attempts over many years, our understanding the mechanisms of activation and inhibition of ACE is still far from complete. Here, we present results of all atom molecular dynamics simulations of ACE with and without ligands. Two types of inhibitors, competitive and mixed non-competitive, were used to model the ligand bound forms. In the absence of a ligand the simulation showed spontaneous large hinge-bending motions of multiple conversions between the closed and open states of ACE, while the ligand bound forms were stable in the closed state. Our simulation results imply that the equilibrium between pre-existing backbone conformations shifts in the presence of a ligand. The hinge-bending motion of ACE is considered as an essential to the enzyme function. A mechanistic model of activation and the inhibition may provide valuable information for novel inhibitors of ACE.

## 1. Introduction

Hypertension, a long-term medical condition known as high blood pressure, is a major risk factor for vision loss, stroke, heart failure and chronic kidney disease, and more than a quarter of the world’s adult population in 2000 had hypertension [1]. Therapeutic approaches have focused on the renin-angiotensin-aldosterone system (RAAS) which regulates blood pressure and electrolyte balance in humans [2]. In the RAAS, renin stimulates the generation of angiotensin I (AngI), which is converted to vasoconstrictor angiotensin II (AngII) by angiotensin I converting enzyme (ACE). Two isoforms (somatic and testicular) of ACE are transcribed by ACE gene in a tissue-specific manner. The somatic form (sACE) is a zinc dependent dicarboxypeptidase, which includes two homologous domains (N domain and C domain) with ~60% sequence identity and the same zinc motif HEXXH (X = any amino acid residue) [3,4]. For the past decades, it has been reported that the C domain of human sACE has the main AngI converting site in controlling blood pressure and cardiovascular functions [5]. ACE hydrolyzes decapeptide (Asp-Arg-Val-Tyr-Ile-His-Pro-Phe-His-Leu) AngI by cleaving a dipeptide from the C-terminus to produce octapeptide (Asp-Arg-Val-Tyr-Ile-His-Pro-Phe) AngII, which causes blood vessels to narrow and stimulates the secretion of the hormone aldosterone, resulting in increased blood pressure [6,7]. ACE also affects blood pressure by inactivating the vasodilators bradykinin and kallidin [8]. Therefore, ACE has long been a major target for the treatment of hypertension and other cardiovascular ailments by the use of ACE inhibitors [9].

Common synthetic ACE inhibitors such as captopril, enalapril, alacepril and lisinopril have been on the market for decades [10]. However, it has been reported for them to have diverse side effects including hyperkalemia and skin rashes [11], impairment of renal function [12], and development of angioedema [13]. Therefore, peptides from natural sources were considered as alternative ACE inhibitors and attracted researchers’ interest [14,15,16,17,18]. The structures of ACE complexes with various ligands including synthetic inhibitors and peptides are available in the Protein Data Bank (PDB).

The crystal structures of ACE have been determined with high resolution, which give us the overall insight of the structural molecular arrangement and their active sites [19,20,21,22]. A crystal structure, which is a time and ensemble averaged snapshot, do not complete our understanding. Proteins are not rigid. They are flexible and dynamic in solution, fluctuating among many conformational sub-states [23]. In addition to its structure, knowledge of the dynamics of an enzyme and understanding the mechanisms of activation and inhibition is crucial for the design of better drugs [24]. Molecular dynamics (MD) simulations are known as a powerful tool for molecular modeling and investigating dynamics of proteins and have been applied to various systems successfully [25]. Recently, MD simulations of ACE with various ligands were reported for drug discovery and molecular modeling for interactions between the enzyme and potential inhibitors [14,15,16,18,26].

**Figure 1:**
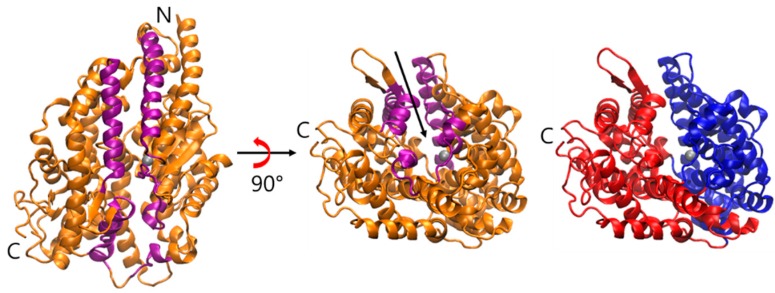
The overview of the structure of C domain of sACE (PDB ID: 4APH). The ribbon representation of sACE shows the secondary structure and the two lips (purple colored) of the mouth. N and C indicate the N- and C-terminus of the enzyme, respectively. Zinc ion is shown as a gray sphere. The rightmost panel shows two subdomains that form two sides of the active site in the cleft, and the subdomain I (residues 40–122, 297–437, 551–583) and II (residues 123–296, 438–550, 584–625) are colored by blue and red, respectively. The arrow indicates the active site near the zinc ion and the putative binding pathway of ligands. The first lip (residues 73–100, 297–304, 348–354, 370–379) belongs to subdomain I, and the second (109–131, 143–156, 267–276) belongs to subdomain II.

In order to explore the ligand binding effects on the dynamics and the mechanisms of activation and inhibition, all-atom MD simulations of sACE were carried out with and without ligands. The simulations focus on the C domain in which the AngI is mainly converted [5]. In the ligand-free simulation (Apo), we observed the mouth opening and closing motions of sACE like the Pac-Man of an arcade game in 1980’s [27]. This large hinge bending motion implies the existence of pre-existing backbone conformations. Figure 1 shows the overall structure of the enzyme. The secondary structure of the sACE is predominantly alpha helical (Figure 1). The structure has an elliptical shape with dimensions approximately 73 × 59 × 52 Å^3^, and the cleft around the active site expands about 31 Å (PDB ID: 4APH). The cleft divides the sACE into two subdomains, and the active site is located on the both of their inner surfaces. Two subdomains of sACE were defined as subdomain I including the N-terminus and the zinc ion (residues Asp40-Leu122, Pro297-Gly437, Asp551-Gly583) and subdomain II including the C-terminus (residues Glu123-Ala296, Gly438-Cys550, Gln584-Ser625). Two kinds of inhibitors, BPPb (bradykinin potentiating peptide b, Glu-Gly-Leu-Pro-Pro-Arg-Pro-Lys-Ile-Pro-Pro) [17] and SPI (*Spirulina* derived heptapeptide, Thr-Met-Glu-Pro-Gly-Lys-Pro) [18] as competitive and mixed non-competitive, respectively, were used to investigate the difference in the inhibition mechanisms. Mixed type non-competitive inhibition mode of SPI was determined by Lineweaver–Burk plot, and a model of inhibition mechanism was studied by the previous study [18].

## 2. Results

### 2.1. Spontaneous Conformational Changes

**Figure 2:**
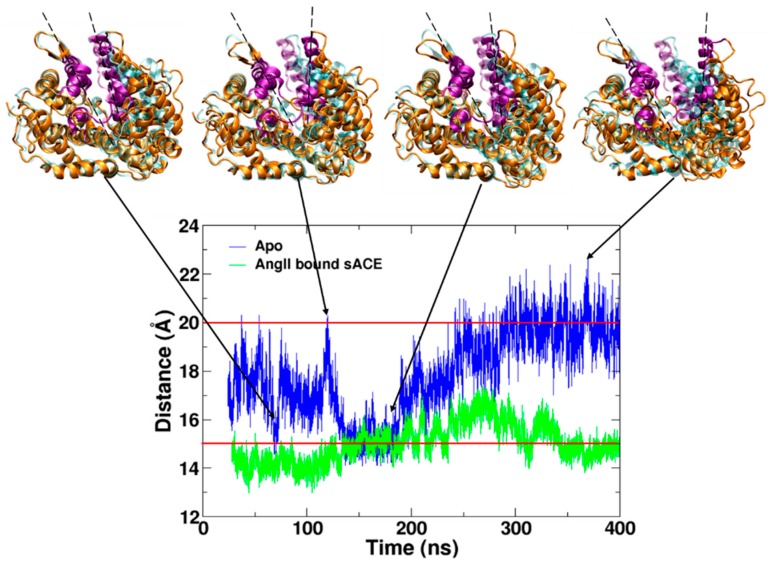
Distance between two lips of AngII bound sACE complex (green) and the Apo form (blue) along the simulation time after discarding the equilibration stage. A conformation with a distance between two lips longer than 20 Å is defined as the open state. With a distance shorter than 15 Å, the conformation is defined as the closed state. If the distance is between 15 and 20 Å, then the conformation is considered as the semi-open state. The snapshots of sACE (orange, purple for lips) are shown by superimposing the subdomain II to the crystal structure (cyan).

A simulation of ligand-free sACE (Apo) was initiated from the coordinates after removing the bound AngII from the sACE-AngII complex (PDB ID: 4APH) [19]. Like all others, the structure of the complex was also in the closed state defined by the distance between two lips (Figure 1) shorter than 15 Å (13.64 Å). As simulation time went by the enzyme spontaneously opened its mouth, and the mouth gradually reclosed from the open state before returning back to the semi-open and open states. We defined the open state with a distance longer than 20 Å and the semi-open state with distances longer than 15 Å and shorter than 20 Å. We observed multiple conversion between the open and closed states during 400 ns simulation (Figure 2). We believe that this is the first work that shows the spontaneous opening and closing motions of ACE by MD simulation (Video S1). In 2019, Yu et al. ran an MD simulation with ligand-free ACE only for 10 ns, but they did not report the opening and closing motions [14].

In order to analyze the mouth opening and closing motion, we defined two lips and calculated the distance between the centers of each lip Cα atoms throughout production stage of the simulations (Figure 2). Two lips of the mouth were defined as lip I in the subdomain I composed of residues Ile73-Arg100, Pro297-Ala304, Arg348-Ala354, Cys370-Val379, and lip II in the subdomain II composed of residues Pro128-Thr150, Gln160-Arg173, Ser284-Phe293. AngII bound sACE was quite stable over the 400 ns simulation time, and no large backbone conformational change was observed unlike the Apo form (in the absence of a ligand). The enzyme mainly stayed in the closed and the semi-open states throughout the entire simulation (Figure S1).

### 2.2. Flexibility of sACE

**Figure 3:**
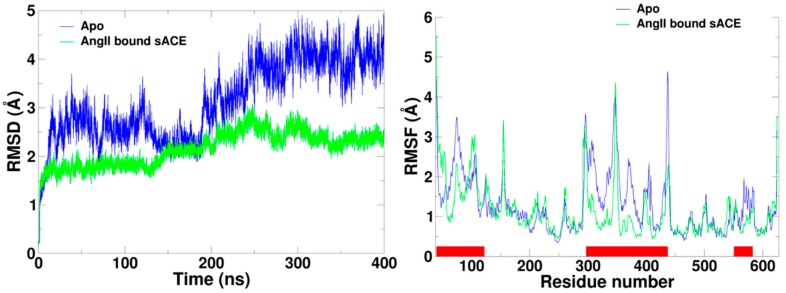
Root-mean-square deviation (RMSD) and root-mean-square fluctuation (RMSF) calculated using Cα atoms from simulations of AngII bound sACE (green) and Apo (blue). The red bars on the horizontal axis of the RMSF graph indicate the residues of subdomain I, showing its flexibility. RMSF was calculated after discarding the equilibration stage of the beginning of the MD simulations.

In order to investigate the flexibility of the enzyme, root-mean-square deviation (RMSD) of the Apo form and the AngII bound form were computed (Figure 3). As we expected the RMSD’s showed the strong correlation with the distances between two lips. Due to the mouth opening motions, the conformation of the Apo form deviated far away from the initial structure, which is in the closed conformation, reaching nearly 5 Å of the RMSD value. However, the RMSD values of AngII bound form were fluctuated less than 3 Å. As compared to the unbound form, the ligand bound form was relatively stable.

Cα root-mean-square fluctuation (RMSF) of each form was calculated using the production stage of the MD simulation trajectories (Figure 3). The analysis indicates that the subdomain I is more flexible than subdomain II for both simulations. Interestingly, the overall flexibility of the subdomain II didn’t change much regardless of the presence of AngII. The most significant difference between the two simulations was the large increment of flexibility on the subdomain I for the Apo form. Notice that the subdomain closure movement in proteins is regarded as a common mechanism for the rearrangement of critical groups around substrates and inhibitors [28].

### 2.3. Open Conformation of ACE2

There is no structural report for the open conformation of human ACE yet. The angiotensin converting enzyme-related carboxypeptidase (ACE2) is a homologue of the human sACE. ACE2 was also identified as the cellular receptor for the SARS coronavirus and novel coronavirus in 2019 (COVID-19) [29,30]. The catalytic domains of ACE2 and sACE share 42% sequence identity [31]. The crystal structures of ACE2 with and without its inhibitor were reported first in 2004 (PDB ID: 1R4L, 1R42). The open conformation was observed in the absence of the inhibitor, while the inhibitor bound one showed the closed conformation. Based on the two structures, the authors proposed a large hinge-bending motion is important for catalytic activity and inhibitor binding of ACE too [32].

We superimposed the closed and the open conformations of ACE and compared with those of ACE2, and the comparison revealed tremendous similarity between two systems (Figure S2). In addition, the distance between two lips was calculated for ACE2. Based on the sequence alignment of ACE2 and sACE, two lips of ACE2 were identified as lip I composed of the residues 54–81, 289–296, 340–346, 361–370 of subdomain I and lip II composed of the residues 109–131, 143–156, 267–276 of subdomain II. The distances between lip I and lip II were computed as 13.32 Å and 20.64 Å for the closed and open states, respectively. This result is consistent with the distance analysis of sACE (Figure 2). In order to test robustness of definition of backbone conformational states we also calculated the mouth open angles, and the results showed the similar pattern as distances (Figure S3). Based on our calculations the maximal hinge-bending movement was ~18° which is close to the value (~16°) measured in ACE2 structures [32].

### 2.4. Competitive Inhibitor Binding

BPPb is a competitive inhibitor isolated from snake venom [17]. A crystal structure shows the active site of sACE occupied by the octapeptide BPPb and the closed conformational state of the complex (PDB ID: 4APJ) [19]. We set up another system with BPPb bound sACE and carried out a 400 ns MD simulation. AngII is not only the product of catalytic function of ACE but also considered as competitive inhibitor. Before leaving the active site, another substrate AngI cannot bind to the site for the next reaction.

**Figure 4:**
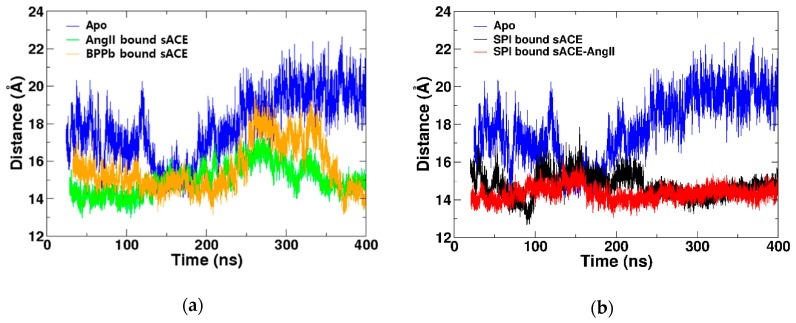
Comparison of the distances between two lips among the Apo and (**a**) competitive inhibitor bound forms with AngII and BPPb. (**b**) Mixed non-competitive inhibitor bound forms with SPI and SPI-AngII complex.

The BPPb bound sACE simulation showed the similar behavior with the AngII bound one (Figure S1). We analyzed the distance between two lips of the mouth, showing conformational changes from the closed to the semi-open states and back to the closed state again (Figure 4a). BPPb bound ACE was more flexible, and the AngII bound one made the mouth closed more tightly. The number of hydrogen bonds between the ligands and the enzyme was calculated over the simulation time (Figure S4). The average numbers were 6.52 ± 2.15 and 6.05 ± 1.64 for AngII and BPPb, respectively (Table 1). This indicated that the opening and closing motions were affected by the interactions between the ligand with the enzyme.

### 2.5. Mixed Non-competitive Inhibitor Binding

In addition to competitive inhibition, non-competitive inhibition is also a common inhibition mechanism. Non-competitive inhibition includes pure and mixed noncompetitive inhibition. Surprisingly for us, mechanism of mixed type non-competitive inhibition for ACE has not been studied intensively so far. In the previous study, a heptapeptide (Thr-Met-Glu-Pro-Gly-Lys-Pro) derived from a marine microalgae *Spirulina* was identified as a mixed non-competitive inhibitor (SPI) [18]. We investigated the interactions and dynamics of ACE bound with the mixed non-competitive inhibitor further. MD simulations were conducted for the SPI bound to the Apo form and the SPI bound to the AngII-sACE complex form.

The simulation of SPI bound to sACE was started with the structure after the removing AngII from SPI bound to AngII-sACE complex, which is the result of the SPI docking simulation of the previous study [18]. The analysis of distance between two lips of the SPI bound sACE showed backbone conformational fluctuation between the closed and semi-open states, but we were not able to see the open conformational state (Figure 4b). In other words, the spontaneous conformational changes were limited, and only the closed and semi-open states were stabilized by SPI (Video S2). The peptide SPI formed 5.86 ± 1.42 hydrogen bonds with sACE averaged over the production stage of the whole simulation (Table 1).

**Figure 5:**
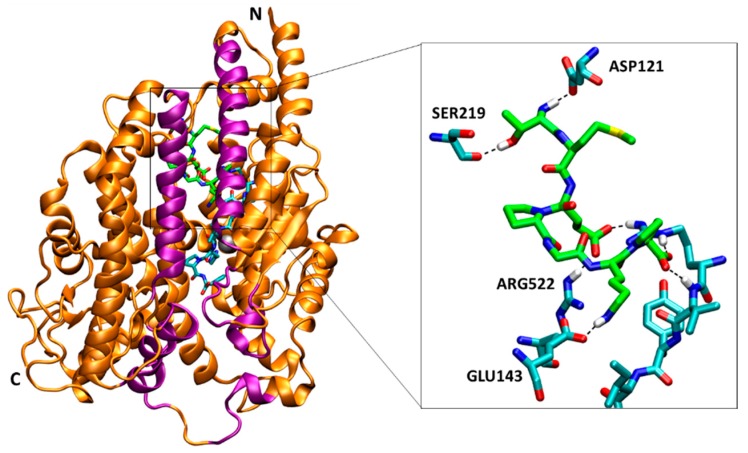
The binding site of the SPI (green carbon) next to AngII (cyan carbon) in the cleft of sACE. sACE is represented by orange ribbons (purple lips), and the zinc ion in the active site is represented by a gray sphere. AngII and ACE binding site are represented by sticks, and carbon, nitrogen, oxygen, sulfur and hydrogen atoms are colored by cyan, blue, red, yellow and white, respectively. SPI is represented by sticks, and carbon atoms are colored by green. Only residue numbers of sACE are shown in the inset figure for clarity. The snapshot was taken at 200.38 ns.

In the simulation of SPI bound to the AngII-sACE complex, the peptide bound in the N-terminal side of the mouth and next to the AngII (Figure 5). Binding of SPI was stabilized by hydrogen bond interactions not only with sACE but also with AngII [18]. We investigated the presence of hydrogen bonds during the MD simulations. In average, the SPI formed 3.22 ± 1.08 and 3.47 ± 0.75 pairs of hydrogen bonds with sACE and AngII, respectively, and together 6.73 ± 1.57 pairs (Table 1). SPI formed hydrogen bonds most frequently with sACE (Thr1-Asp121, Thr1-Ser219, Glu3-Arg522, Lys6-Glu143, SPI-sACE residues in order) and with AngII (Glu3-Arg2, Pro7-Arg2, Pro2-Val3, SPI-AngII residues in order) (Figure 5, Table S1). In addition to hydrogen bonds, binding of the SPI was stabilized by van der Waals interactions too. The sidechain of Pro4 of the SPI bound in the hydrophobic pocket formed by Ile204, Leu139 and Trp220 of sACE (not shown in Figure 5 for clarity).

Distance analysis of SPI bound sACE-AngII showed similar behavior to other ligand bound simulations (Figure 4b). Interestingly, the SPI bound sACE-AngII complex was stayed in the closed state most of the simulation time. This is understandable since both SPI and AngII are acting as inhibitors, and the number of hydrogen bonds between the ligand and the enzyme is largest among all complexes (Table 1). The closed state was stabilized not only by the interactions between AngI and sACE but also by the interactions between SPI and sACE. In addition, interactions between SPI and AngII stabilized the complex further in the closed state. Analysis of all five RMSF’s indicated that the most stable system was SPI bound to sACE-AngII complex (Figure S5). We recognized that the binding of SPI to sACE-AngII complex slightly twisted the subdomain I (Figure S6).

## 3. Discussion

The active sites and binding interactions have been well known for ACE with various inhibitors, while the conformational dynamics and its role in activation mechanism have not been studied intensively. Drug design might benefit from backbone conformations observed by MD simulations, which have never reached by the X-ray crystallography with ligand bound forms. Unlike the closed state, the open state of sACE has not been detected in both experiments and simulations so far. Surprisingly, over more than a decade since the first proposal [32], no further study has been reported about the role of large hinge-bending motion for the activation of sACE.

**Figure 6:**
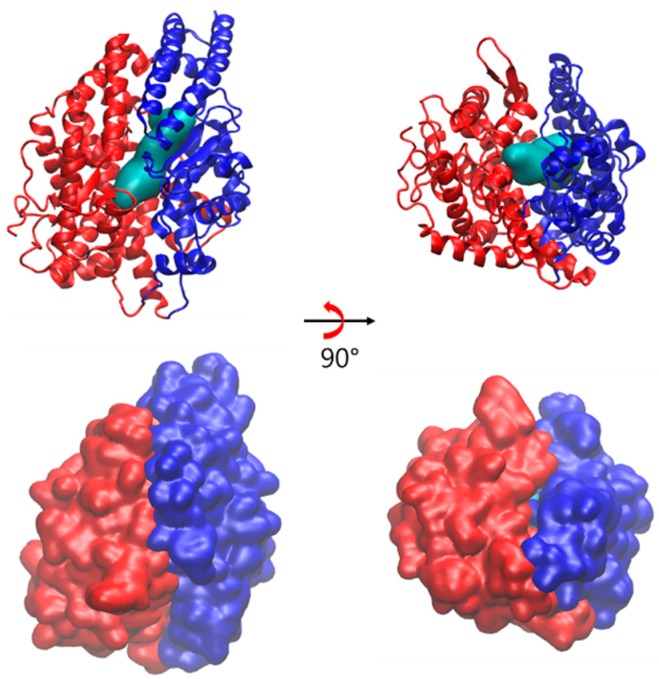
Surface representations of AngII (cyan colored) and ribbon representation of a closed state of sACE (subdomain I in blue and subdomain II in red) at the top panel and its surface representations of sACE at bottom panel.

In contrast to the lock-and-key model, many proteins can take a ligand only through the conformational changes. The putative binding pathway of sACE might be too narrow to accommodate the substrate AngI in the closed state (Figure 6), thus the enzyme needs to change its backbone conformation to take the substrate for its catalytic function. At least in the semi-open state, rearrangement of sidechain may allow the substrate to enter the active site (Figure S7). Two models of enzyme mechanisms, induce-fit [33] and pre-existing equilibrium dynamics [34] represent the conformational changes and dynamics upon ligand binding. In the former, binding of a ligand induces conformational changes of a protein, while in the latter, a ligand binds to a certain pre-existing conformation already present and accessible by equilibrium dynamics.

In the ligand-free (Apo) MD simulation, we were able to observe spontaneous conformational changes between the open and closed states of sACE, implying that the open and closed backbone conformational states are already present and accessible regardless of the presence of a ligand. In addition, several times of conversions between the states for the 400-nanoseconds simulation indicate that the enzyme is very flexible and dynamic. Indeed, all available ACE structures are resolved in the closed conformation either in the presence ligands or with mutations. Introducing mutations, ligands or chimera are common methods to enhance thermal stability and to crystalize a protein. In addition, the absence of the open conformation in a ligand-free ACE imply the intrinsic flexibility of ACE. Again, only one available structure in the open conformation is resolved with ACE2 [32]. In addition, a previous study using normal mode analysis (NMA) with testicular ACE (tACE, another isoform of ACE) and ACE2 showed the intrinsic flexibility and open and closed conformational models of ACE [35]. The authors also proposed the hinge-bending mechanism for substrate entry into the active site.

**Figure 7:**
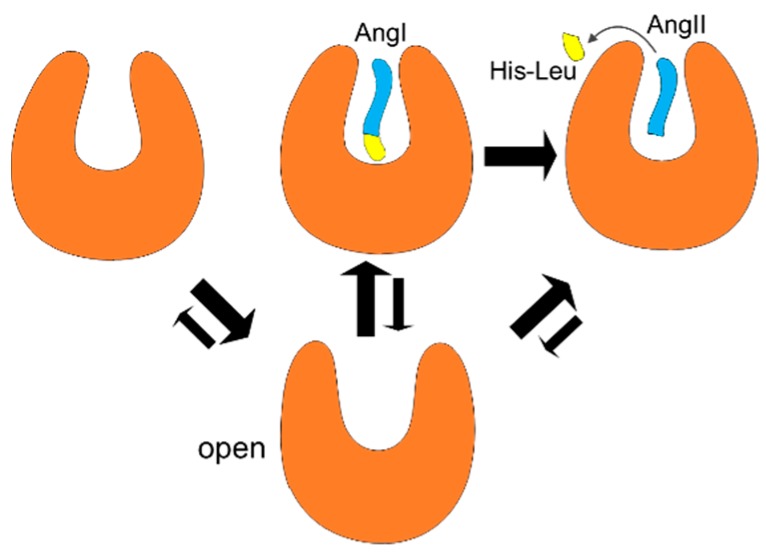
Activation mechanism of sACE and equilibrium shifts between the open conformation (lower panel) and closed conformations (upper panel) upon the presence of ligands: from the left to right in the figure, in the absence of a substrate (AngI), in the presence of substrate, and in the presence of product (AngII), respectively. Equilibrium shifts between two states are represented qualitatively by the size of arrows.

Based on the results of our MD simulations and available X-ray structures, we propose the following activation mechanism of sACE (Figure 7). Regardless of presence of a ligand, sACE could be in any of the open, semi-open and closed states in terms of backbone conformation. In the absence of a substrate, or AngI, the open state might be more favored than the closed state (Figure 2), and the enzyme becomes ready to accept a substrate for its catalytic function. This equilibrium, however, is changed in the presence of AngI, and the closed state becomes more stable than the open state due to the binding of the substrate. Then the C-terminal dipeptide (His-Leu) of AngI is hydrolyzed. AngII, the product of the enzyme activity, might be released when sACE is in the semi-open state (Figure 2 and Figure S7). After the release of AngII from sACE, the equilibrium between conformational states goes back to the first stage again. Notice that the semi-open conformation can be accessed under any condition.

Similarly, we can derive a mechanism for a competitive inhibitor. Now, if the ligand is a competitive inhibitor, then the bound complex becomes stable in the closed state. Qualitatively, the equilibrium shift is the same way as the case of substrate binding. After release of the inhibitor, the equilibrium goes back to the first stage of the Apo form. Since AngII occupies the same active site, until the release of AngII sACE cannot take any substrate. This is true for the substrate too. Interestingly, SPI the mixed non-competitive inhibitor makes use of this. The interactions of SPI not only with sACE but also with AngII stabilized sACE in the closed state further, resulting in a dead-end complex by holding the product of enzyme AngII in the active site [18]. Since SPI does not share the binding site with the substrate, it becomes non-competitive. Even though SPI is non-competitive, it binds next to the active site and interacts with the substrate, becoming a mixed type inhibitor [18]. The binding sites of various drugs and drug candidates were studied intensively, and along the various ligands, binding sites and their conformations are various too. This indicates the binding of a specific ligand induce a specific conformation of its binding site. Therefore, the overall large backbone open and closed conformational state pre-exist, and the sidechain and minor backbone rearrangement may be induced upon ligand binding.

In Apo simulation, we have removed the bound AngII from the X-ray structure of sACE-AngII complex and started the MD simulation. This removal of AngII is an additional perturbation to the sACE structure. We checked equilibration of the Apo system by monitoring the thermodynamic parameters such as energy, temperature, volume and pressure (Figure S8). In addition, we monitored evolution of secondary structure along the simulation time and recognized that the overall secondary structure was well conserved and stable except for loop and turn regions (Figure S9). Even though we observe multiple opening and closing motions, one can still consider the Apo system is still under equilibration stage. If Apo structure was still under equilibration stage and goes to a certain open conformation, then we would have an X-ray structure with open conformation like ACE2. A calorimetric study showing that the drug binding is entropically driven also supports dynamics of ACE [36]. 

In conclusion, using all-atom MD simulations, the conversion between open and closed states of sACE was observed in the absence of a ligand supporting that hinge-bending motion is essential in enzyme activation. The preferred states as well as the extent of flexibility were strongly dependent on the presence of a ligand. The transformation from the semi-open to the closed state in the bound forms was directly influenced by the interaction between the residues of binding sites of the substrate and the inhibitors. These interactions constrained the enzyme structure to remain in the semi-open and closed states. However, it would seem that the putative binding pathway is too narrow to accommodate the substrate and inhibitors. Therefore, the semi-open conformation might be the state where the ligands can bind. Our MD simulation results and the mechanistic model of sACE activation and inhibition portends not only drug design for hypotension treatment but also wide applications in molecular biophysics and beyond.

## 4. Materials and Methods

### 4.1. Molecular Docking for SPI

Potential binding sites of SPI on sACE were searched by docking simulations using the FlexPepDock protocol of the Rosetta program [37]. The crystal structure of human sACE in complex with AngII (PDB ID: 4APH) was used for the target. Since the SPI peptide is a non-competitive inhibitor, AngII was kept during the docking simulations to avoid overlapping between the binding site of the peptide and the active site. A structure with the lowest score of docking simulations was chosen for the molecular dynamics simulation in order to relax the structure further (Table S2). Details of the docking simulations were described in the previous study [18].

### 4.2. Preparation of MD Simulations

Total five molecular dynamics simulations were carried out: BPPb bound sACE, AngII bound sACE, Apo (ligand-free), SPI bound sACE-AngII complex, SPI bound sACE. Two crystal structures of the sACE co-crystallized with ligands in the active site were used to build the simulation systems, sACE complex with the natural inhibitory peptide BPPb (PDB ID: 4APJ) and sACE complex with AngII (PDB ID: 4APH). In order to simulate the Apo form, AngII was removed from the active site of the crystal structure (4APH). The SPI bound to the sACE-AngII complex with the lowest score from the docking simulations was chosen to run the fourth MD simulation, and the last SPI bound sACE model was generated by removing the AngII from this docking simulation result.

Charmm36 force field with explicit TIP3P water molecules was used in all simulations [38]. Crystallographic water molecules, chloride ions, and zinc ion were retained as they were found in the X-ray crystal structures. Ligand chemical components in the original crystal structures including acetate ion, beta-D-mannose, N-acetyl-D-Glucosamine were also retained and modeled with Charmm general force field [39]. All five simulation systems were solvated in the truncated octahedral box with 23251, 23418, 23565, 23410 and 23374 TIP3P water molecules, respectively. Then these solvated systems were electrically neutralized by adding counter ions, and the final salt concentration became 0.15 M.

### 4.3. Molcular Dynamics Simulations

All simulations were performed by NAMD under the periodic boundary condition [40]. After solvation, 1000 steps of energy minimization were performed to remove possible bad contacts. Then, the MD simulations were started by gradually heating the systems from 10 to 310 K for 60 ps under the constant volume condition. In order to equilibrate the density of the systems, the simulation was switched to constant pressure and temperature (NPT) conditions and continued afterwards. The average pressure and the temperature were maintained at 1 bar and 310 K, respectively. All heavy atoms during the energy minimization, heating and the first 200 ps of NPT simulation were restrained with harmonic potential with a force constant 1 kcal/mol·A^2^. After that, the harmonic position restraints were removed, allowing all atoms in the systems to relax. All bonds involving hydrogen atoms were constrained, allowing an integration time step of 2 fs. The non-bonded interactions were smoothly truncated from 10 Å to 12 Å cutoff, and the particle-mesh Ewald method was used to treat long-range electrostatic interactions [41]. The equilibration stages of simulations were determined based on RMSD and system energy calculations. Total simulation and equilibration times for BPPB-sACE, AngII-sACE, Apo, SPI-sACE-AngII, and SPI-sACE were 400 (32.26), 400 (28.26), 400 (24.36), 360 (20.95), and 360 (13.26), respectively in nanoseconds. Summary of all MD simulations are provided in Table S3. The production stage after discarding the initial equilibration stage, was used for the post simulation analysis except for RMSD calculation. All post analyses and visualization were carried out by Visual Molecular Dynamics (VMD) program [42]. Formation of hydrogen bond was identified using 3.3 Å (donor-acceptor distance) and 30 degrees (proton-donor-acceptor angle) criteria.

## Figures and Tables

**Table 1:** The average number of hydrogen bonds between the ligand and the enzyme calculated over the production stage of molecular dynamics (MD) simulations.

Complex	Average Number of Hydrogen Bond
AngII bound sACE	6.52 ± 2.15
BPPb bound sACE	6.05 ± 1.64
SPI bound sACE	5.86 ± 1.42
SPI bound sACE-AngII complex	6.73 ± 1.57

## Supplementary Materials

The following are available online at https://www.mdpi.com/1420-3049/25/6/1288/s1, Figure S1: Conformational states of sACE from MD simulations, Figure S2: Superposition of the closed state and the open state of sACE and ACE2, S3: Mouth open angles, S4: Number of hydrogen bonds along the simulation, S5: Cα RMSF of all simulation systems, S6: SPI induced conformational change, S7: Semi-open state, S8: Equilibration of Apo simulation, S9: Diagram of secondary-structure evolution, Table S1: Ratio of forming hydrogen bond pairs over the simulation time, S2: Major docking score functions, Table S3: Summary of MD simulations, Video S1: Mouth opening of Apo sACE, Video S2: SPI bound sACE.
