# Erratum: Phan, T.T.V., et al. Spontaneous Hinge-Bending Motions of Angiotensin I Converting Enzyme: Role in Activation and Inhibition. *Molecules* 2020, *25*, 1288

**DOI:** 10.3390/molecules26061508

**Published:** 2021-03-10

**Authors:** Thi Tuong Vy Phan, Seong-Yeong Heo, Won-Kyo Jung, Myunggi Yi

**Affiliations:** 1 Center for Advanced Chemistry, Institute of Research and Development, Duy Tan University, 03 Quang Trung, Hai Chau, Danang 550000, Vietnam; phanttuongvy4@duytan.edu.vn; 2 Interdisciplinary Program of Biomedical, Electrical & Mechanical Engineering, Pukyong National University, Busan 48513, Korea; hsyadsl@naver.com (S.-Y.H.); wkjung@pknu.ac.kr (W.-K.J.); 3 Department of Biomedical Engineering, Pukyong National University, Busan 48513, Korea; 4 Marine Integrated Bionics Research Center, Pukyong National University, Busan 48513, Korea

The authors wish to make the following corrections to the paper [1]: During the final preparation of the manuscript, the family name of the first author was omitted; also, the affiliation and the e-mail of the first author were not correctly input. We would thus like to make following changes to the paper [1], as follows:

**Thi Tuong Vy Phan ^1^**

^1^  Center for Advanced Chemistry, Institute of Research and Development, Duy Tan University, 03 Quang Trung, Hai Chau, Danang 550000, Vietnam; phanttuongvy4@duytan.edu.vn

We apologize for this unintentional mistake, which, however, does not affect the results of this manuscript and the conclusions drawn from them.
