# Supplementary material for: Spontaneous Hinge-Bending Motions of Angiotensin I Converting Enzyme: Role in Activation and Inhibition
Source: Molecules. 2020 Mar 12;25(6):1288. doi: 10.3390/molecules25061288 (PMC7146279; doi:10.3390/molecules25061288)
Supplement: Supplementary file 1 [file molecules-25-01288-s001.zip › ACEsupplementary_rev.pdf]

Spontaneous hinge-bending motions of angiotensin I converting enzyme: Role in activation and inhibition

Thi Tuong Vy [1], Seong-Yeong Heo [1,2], Won-Kyo Jung [1,2,3], and Myunggi Yi [1,2,*]

[1] Interdisciplinary Program of Biomedical, Electrical & Mechanical Engineering, Pukyong National University, Busan 48513, Republic of Korea; phanvy120690@gmail.com (T. V.); hsyadsl@naver.com (S-Y. H.);

[2] Department of Biomedical Engineering, Pukyong National University, Busan 48513, Republic of Korea; myunggi@pknu.ac.kr

[3] Marine Integrated Bionics Research Center, Pukyong National University, Busan 48513, Republic of Korea; wkjung@pknu.ac.kr

* Correspondence: myunggi@pknu.ac.kr; Tel.: +82-51-629-5773

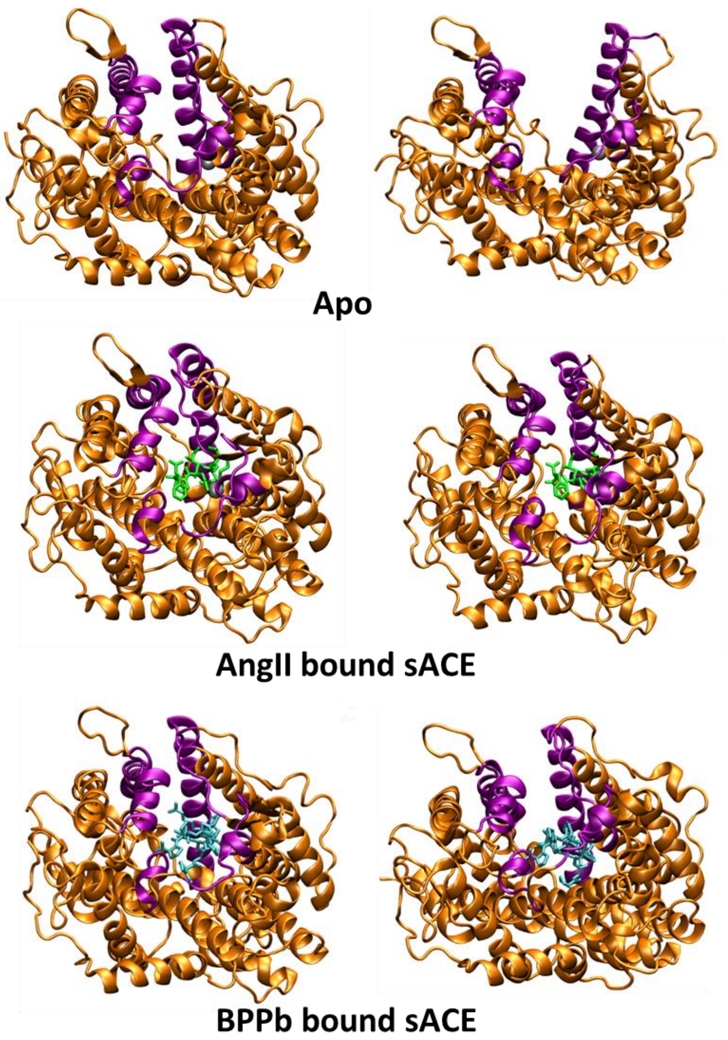

Figure S1: Backbone conformational states of sACE from MD simulations. Backbone Conformational states of sACE are represented by orange ribbons with purple lips, and the ligands are represented by sticks. From the top left, semi-open and open states of Apo, closed and semi-open states of AngII bound form, closed and semi-open states of BPPb bound form are shown. The snapshots were taken at 269.86 ns and 369.65 ns of the simulation time of Apo, at 400 ns and 277.6 ns of the simulation time of AngII bound sACE, and at 400 ns and 270 ns of the simulation time of BPPb bound sACE.

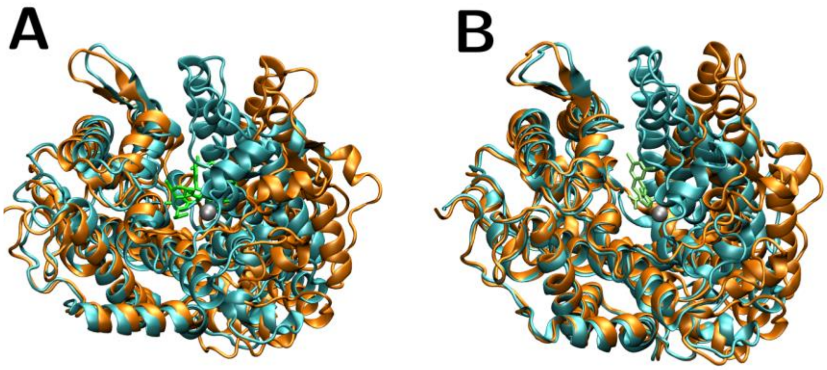

Figure S2: Superposition of the closed state and the open state of sACE and ACE2. (A) An open conformational state of Apo is superimposed to the closed conformational state of AngII bound (crystal structure, 4APH) sACE. (B) The open state of ligand-free ACE2 (PDB ID: 1R42) is superimposed to the MLN-4760 inhibitor bound (PDB ID: 1R4L) ACE2. Superposition was performed using Cα atoms of subdomain II. The open states and closed states are colored by orange and cyan, respectively. Ligands are represented by green sticks.

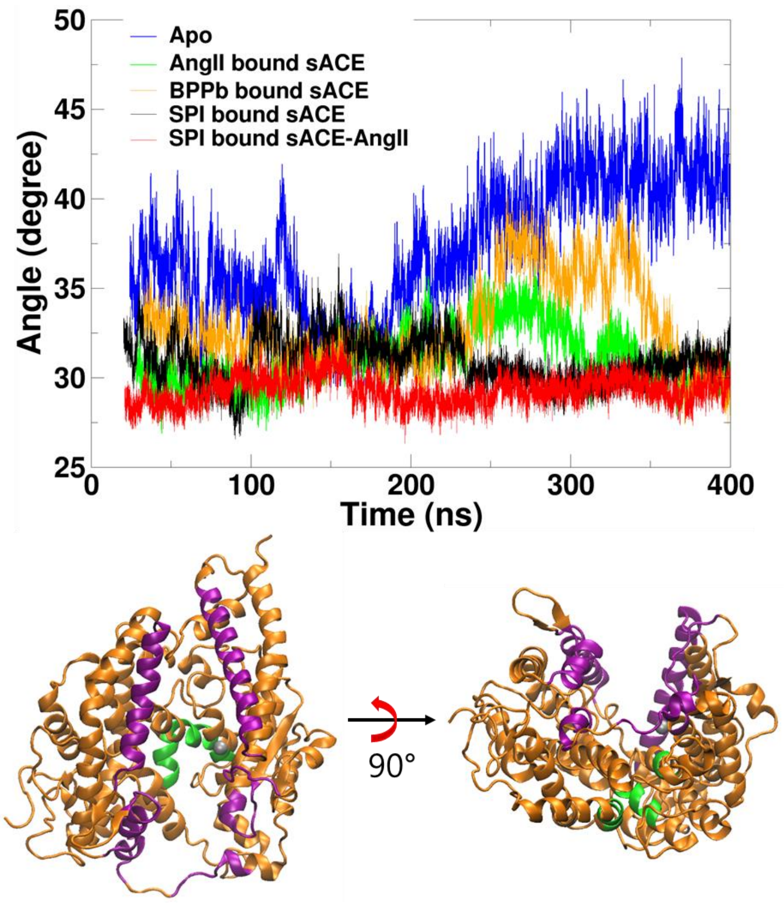

Figure S3: Mouth open angles calculated along the simulations (top panel). Angles are calculated by three points. Two points are the centers of two selections of lips (puple colored in the bottom panel). In addition to lips, we selected residues 415-417, 529-535, 591-596 as the axis (green colored in the bottom panel) of bending motions. Residues of axis are selected by geometric consideration for angle calculations and by stability consideration with lower than 0.8 Å of RMSF values. Each center of selections is calculated by geometric center of Cα atoms. The patterns of angles are almost same as those of distances. The maximal hinge-bending movement of Apo was ~18°.

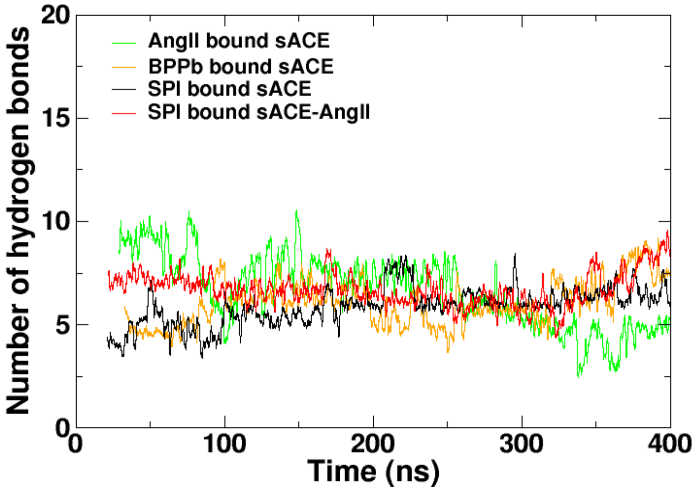

Figure S4: The numbers of Hydrog bonds between ligands and sACE and sACE-AngII complex along the simulation time. Due to the large and frequent fluctuations of the numers, running averages with 100-data size are displayed for clarity and comparison.

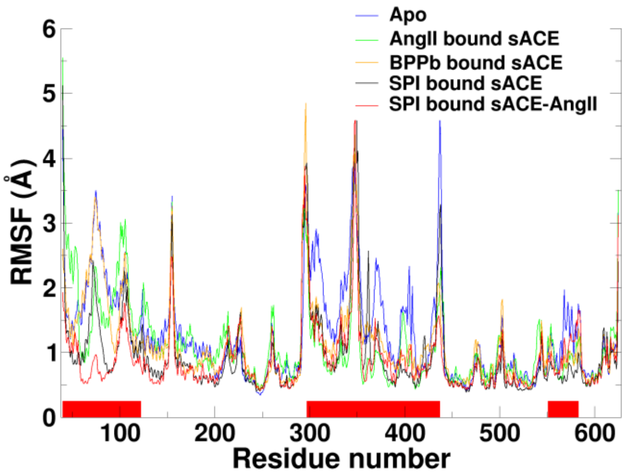

Figure S5: Cα RMSF of all simulation systems. The RMSF's were calculated after discarding the equilibration stage of MD simulations. The most significant difference among simulations is a large increased flexibility of the subdomain I for the Apo for, and the most stable system is the SPI bound to sACE-AngII complex.

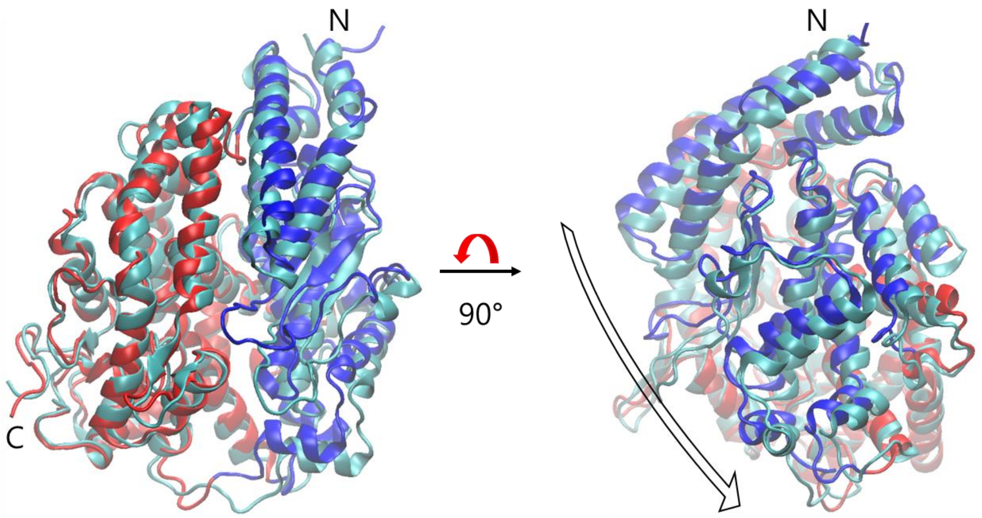

Figure S6: SPI induced conformational change into sACE-AngII complex. Subdomain I of SPI bound sACE-AngII complex (cyan) is slightly twisted clockwise compared to that of sACE-AngII complex (blue). Two structures are superimposed by using Cα atoms of subdomain II (red). Both subdomains of SPI bound sACE-AngII complex are colored by cyan.

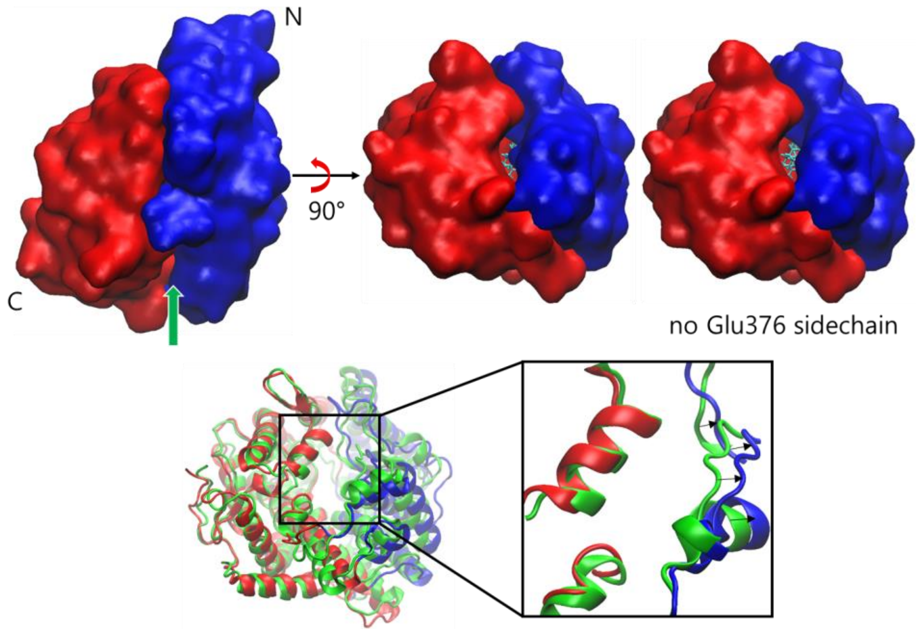

Figure S7: A semi-open state of AngII bound sACE. Subdomains (I in blue, II in red) and AngII (cyan) are represented by surfaces and sticks, respectively. A possible entrance in the semi-open state is indicated by a green arrow. The snapshot (300 ns) was taken from the AngII bound sACE simulation, and the distance between two lips was 15.4 Å. The bottom panel shows that a major difference between

the closed (green, at 0 ns) and semi-open (red and blue) states is around the C-terminal side of the mouth (superposition by Cα atoms of subdomain II). At least in semi-open state, the substrate may gain access to the entrance to the active site by rearrangement of sidechain (rightmost of top panel).

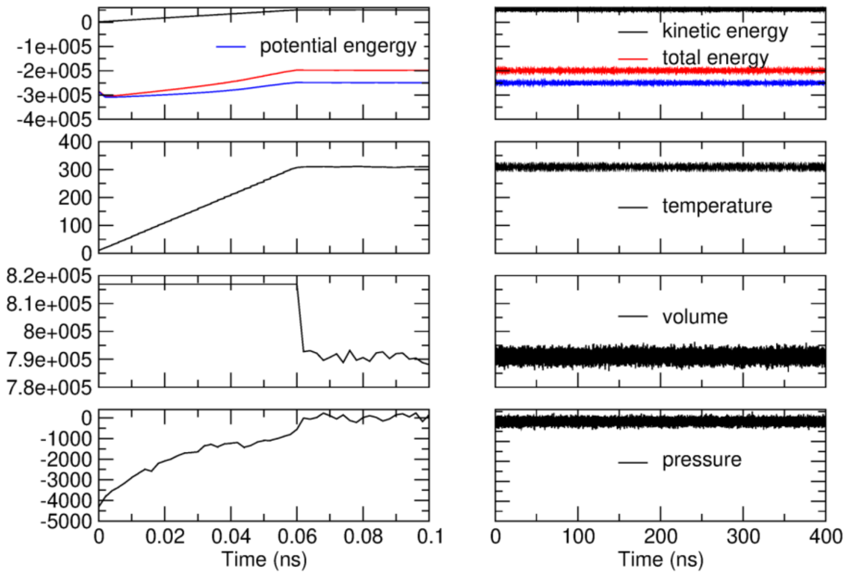

Figure S8: Equilibration of Apo simulation system. Thermodynamic parameters along the simulation time is on the right column. Data during the first 0.1 ns of the simulation are plotted separately on the left column for clarity with different time scale.

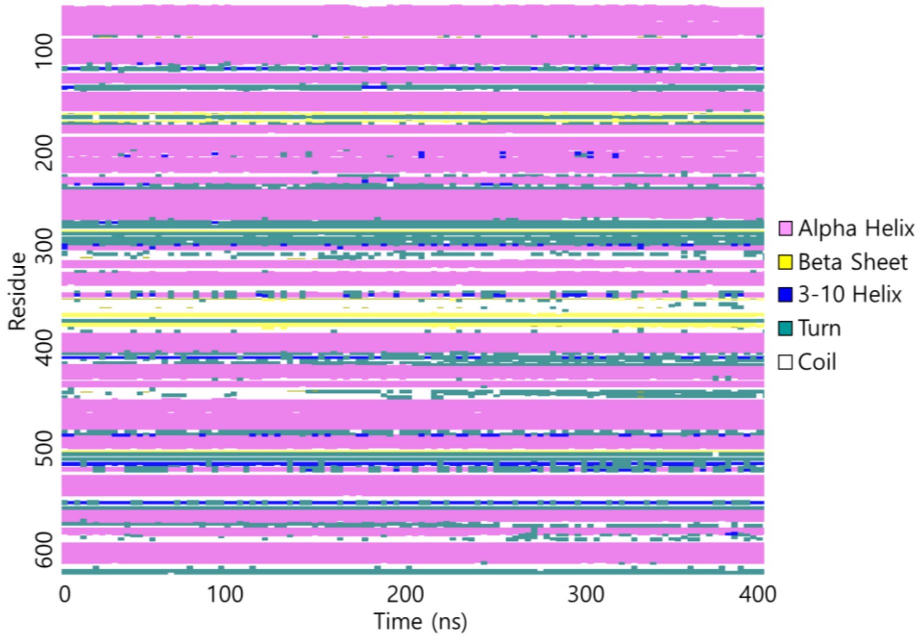

Figure S9: Diagram of secondary-structure evolution of sACE along the Apo simulation time.

Table S1: Ratio of forming hydrogen bond pairs over the simulation time.

SPIand AngII	Ratio (%)
Glu3 - Arg2	98.00
Pro7 - Arg2	87.25
Pro7 - Val3	81.03
**SPI and sACE**	**Ratio (%)**
Glu3 - Arg522	85.88
Thr1 - Ser219	69.19
Thr1 - Asp121	58.75
Lys6 - Glu143	57.23

Table S2: Major docking score (energy) functions, brief description and scores of the lowest scored model.

Score function	Score	description
fa_atr	-2590.578	Lennard-Jones attractive
fa_rep	404.603	Lennard-Jones repulsive
fa_sol	1254.254	Solvation
fa_elec	-50.706	Coulombic electrostatic
hbond	-367.233	Sum of hydrogen bond scores
omega	78.526	Omega dihedral in the backbone
fa_dun	510.233	Sidechain rotamers

Table S3: Summary of MD simulations.

Name	Total length (ns)	Equilibration stage to (ns)	Starting structure	Initial conformation	Sampling conformations
AngII bound sACE	400	28.26	PDB ID: 4APH	closed	closed, semi-open
Apo	400	24.26	AngII removed 4APH	closed	closed, semi-open, open
BPPb bound sACE	400	32.26	PDB ID: 4APJ	closed	closed, semi-open
SPI bound sACE-AngII complex	400	20.95	SPI bound sACE-AngII complex (4APH)	closed	closed, semi-open
SPI bound sACE	400	20.26	AngII removed SPI bound sACE-AngII complex	closed	closed, semi-open

Video S1: Mouth opening of Apo sACE.

Video S2: SPI bound sACE.
